# Investigation of Spectroscopic Properties and Spin-Orbit Splitting in the X^2^Π and A^2^Π Electronic States of the SO^+^ Cation

**DOI:** 10.3390/ijms13078189

**Published:** 2012-07-03

**Authors:** Wei Xing, Deheng Shi, Jinfeng Sun, Zunlue Zhu

**Affiliations:** College of Physics and Information Engineering, Henan Normal University, Xinxiang 453007, China; E-Mails: wei19820403@163.com (W.X.); jfsun@htu.cn (J.S.); zl-zhu@htu.cn (Z.Z.)

**Keywords:** potential energy curve, spin-orbit coupling, spectroscopic parameter, scalar relativistic correction, core-valence correlation correction

## Abstract

The potential energy curves (PECs) of the X^2^Π and A^2^Π electronic states of the SO^+^ ion are calculated using the complete active space self-consistent field method, which is followed by the internally contracted multireference configuration interaction (MRCI) approach for internuclear separations from 0.08 to 1.06 nm. The spin-orbit coupling effect on the spectroscopic parameters is included using the Breit-Pauli operator. To improve the quality of PECs and spin-orbit coupling constant (*A*_0_), core-valence correlation and scalar relativistic corrections are included. To obtain more reliable results, the PECs obtained by the MRCI calculations are corrected for size-extensivity errors by means of the Davidson modification (MRCI+Q). At the MRCI+Q/aug-cc-pV5Z+CV+DK level, the *A*_0_ values of the SO^+^(X^2^Π_1/2, 3/2_) and SO^+^(A^2^Π_1/2, 3/2_) are 362.13 and 58.16 cm^−1^ when the aug-cc-pCVTZ basis set is used to calculate the spin-orbit coupling splitting, and the *A*_0_ of the SO^+^(X^2^Π_1/2, 3/2_) and SO^+^(A^2^Π_1/2, 3/2_) are 344.36 and 52.90 cm^−1^ when the aug-cc-pVTZ basis set is used to calculate the spin-orbit coupling splitting. The conclusion is drawn that the core-valence correlations correction makes the *A*_0_ slightly larger. The spectroscopic results are obtained and compared with those reported in the literature. Excellent agreement exists between the present results and the measurements. The vibrational manifolds are calculated, and those of the first 30 vibrational states are reported for the *J* = 0 case. Comparison with the measurements shows that the present vibrational manifolds are both reliable and accurate.

## 1. Introduction

The SO^+^ ion is an important species of considerable physical, chemical and astrophysical interest. The ion is isovalent to O_2_^+^ and is one of the main constituents of plasmas containing sulfur and oxygen. In the past several decades, it has been detected in interstellar molecular clouds [1–3], the plasma torus of Jupiter [4], comet Halley [5] and the Io torus [6–8]. Its concentration may be a critical indicator of the chemistry of both the plasma torus surrounding Jupiter in the orbit of Io [4] and the interstellar clouds. Besides these, in the ion chemistry of the Earth atmosphere, the role of the cation is also very important. At the same time, its spectral information is of great significance in scientific experiments and material analyses [9]. Therefore, it is not surprising that a lot of attention has been paid to the spectroscopic and molecular properties of the ion not only by experimental methods, but also theoretically as well.

Laboratory spectroscopic studies of the SO^+^ cation have been undertaken for more than three decades. The first observations were made by Dyke *et al*. [10] in 1974, who characterized the SO^+^ ion by vacuum ultraviolet photoelectron spectroscopy (PES). Dyke *et al*. [10] determined the potential energy curves (PECs) of six electronic states, but failed to locate the origins of the A^2^Π and a^4^Π due to impurities. Next, Tsuji *et al*. [11] in 1980 observed the extensive bands from the helium afterglow reaction of SO_2_ in the 250–540 nm region and assigned these transitions to the SO^+^(X^2^Π-A^2^Π) band system. Shortly after in 1982 [12], they determined the absolute vibrational quantum numbers for the SO^+^(X^2^Π-A^2^Π) emission system by measurement of isotopic shifts between the S^16^O^+^ and the S^18^O^+^ bands. Cossart *et al*. [13] in 1983 made the rotational analysis for the first time for the X^2^Π-A^2^Π as well as b^4^∑^−^-a^4^Π band systems of the SO^+^ cation, and determined some accurate spectroscopic parameters of the four electronic states, X^2^Π, A^2^Π, a^4^Π and b^4^∑^−^. In a parallel study, Coxon and Foster [14] in 1984 recorded nine bands of the A^2^Π→X^2^Π band system. Hardwick *et al*. [15] in the same year recorded 0–5, 0–6, 1–5 and 1–6 bands of the A^2^Π-X^2^Π band at high resolution. The corresponding rotational analyses were also made in their work [14,15]. A number of spectroscopic parameters and molecular constants were determined for the two electronic states in these investigations [10–15].

Subsequently, Milkman *et al*. [16] in 1986 recorded the A^2^Π-X^2^Π band system of the cation in a rotationally cold supersonic expansion at a resolution of 0.3 cm^−1^ and made some rotational analyses. The derived constants for this band system and reported for 60 bands involving *υ*″ = 0–10 and *υ*′ = 0–11. Then in 1988, they [17] observed rotationally cold emission for the A^2^Π-X^2^Π band of the SO^+^ cation at high resolution using a slot-shaped corona excited supersonic expansion. Bands with *υ*′ = 0–8 and *υ*″ = 3–11 have been assigned and analyzed at high resolution. The spectroscopic results they obtained are of high quality to this day. Norwood and Ng [18] in 1989 measured photoion-photoelectron coincidence (PIPECO) spectra in the wavelength range from 102.5 to 121.0 nm for the SO and S_2_O molecules by a pulsed PIPECO approach. Vibronic bands attributable to the formation of the SO^+^ (X^2^Π_3/2, 1/2_, *υ* = 0–11) were resolved in the SO^+^ PIPECO spectra. Amano *et al*. [19] in 1991 observed the rotational transitions in the ^2^Π_3/2_ electronic state, and obtained a more complete set of spectroscopic parameters, including the effective spin-rotation coupling constant. Dyke *et al*. [20] in 1997 reported their PES measured by the vacuum ultraviolet radiation from a synchrotron. Some spectroscopic parameters and molecular constants of the involved electronic states were determined. Recently, Li *et al*. [21] in 2008 recorded the absorption spectrum of the fundamental band of the SO^+^(X^2^Π) cation using a mid-infrared tunable diode laser spectrometer with the velocity modulation technique in an AC glow discharge of He/SO_2_, and identified forty-two lines of the SO^+^ cation in the spectral range from 1230 to 1330 cm^−1^. As seen in the experimental literature [16–21], a number of spectroscopic parameters and molecular constants were also obtained.

In the past more than thirty years, a number of experiments [10–15,17,18,20,22] have been made to determine the spin-orbit coupling constant (*A*_0_) of the SO^+^(X^2^Π_1/2, 3/2_) and SO^+^(A^2^Π_1/2, 3/2_). Of these experiments, the first one was made by Dyke *et al*. [10] in 1974, who measured the experimental *A*_0_ value, 340 ± 10 cm^−1^ for the SO^+^(X^2^Π_1/2, 3/2_) using photoelectron spectrometer. Then, Tsuji *et al*. obtained *A*_0_ values of 414 ± 5 in 1980 [11] and 412 ± 13 cm^−1^ in 1982 [12], respectively. As to the *A*_0_ result of the SO^+^(A^2^Π_1/2, 3/2_), the first one was reported by Cossart *et al*. [13], who determined the experimental *A*_0_ value of 72 cm^−1^ in 1983. Meanwhile, they [13] also reported the *A*_0_ value of 352 cm^−1^ for the SO^+^(X^2^Π^1/2, 3/2^). Among the theoretical values for *A*_0_, the following two are considered to be of the highest quality: one determined by Lam *et al*. [22] in 2011 and the other reported by Milkman *et al*. [17] in 1988. Lam *et al*. [22] obtained the *A*_0_ value of 365.26 cm^−1^ for the SO^+^(X^2^Π^1/2, 3/2^). Milkman *et al*. [17] determined the *A*_0_ values of 364.38 for the SO^+^(X^2^Π_1/2, 3/2_) and 53.880 cm^−1^ for the SO^+^(A^2^Π_1/2, 3/2_).

The first theoretical work on the SO^+^ cation could be traced back to that of Dyke *et al*. [10] in 1974. Dyke *et al*. calculated the spin-orbit splitting for the SO^+^(X^2^Π_1/2, 3/2_) using the wave functions obtained by the restricted complete neglect of differential overlap (CNDO) calculations. The first *ab initio* work on the SO^+^ cation was reported by Cossart *et al*. [13] in 1983. Cossart *et al*. [13] made the spectroscopic parameter calculations for the X^2^Π and A^2^Π electronic states employing the self-consistent field (SCF) method followed by the configuration interaction (CI). Klotz *et al*. [23] in the same year studied the zero-field splitting for the ground state of the cation using the standard multireference CI (MRD-CI) method, and calculated the *A*_0_ values using two groups of atomic orbit (AO) basis sets. Balaban *et al*. [24] in 1989 optimized the structures of 12 molecules. For the SO^+^(X^2^Π) cation, they determined its *R_e_* value of 0.1411 nm at the SCF/6-31G*(5*d*) level. Midda and Das [25] in 2003 studied the molecular properties of the SO^+^(X^2^Π) cation using the hybrid density functional HF/DF B3LYP method and four basis sets from 6-311++G(2*df*, 2*pd*) to aug-cc-pVTZ. They determined its *R_e_* value to be 0.1421 nm. More recently, Houria *et al*. [9] in 2006 made the spectroscopic and spin-orbit coupling calculations on the SO^+^ cation. Favorable agreement with the measurements has been found. Very recently, Lam *et al*. [22] in 2011 made high-level *ab initio* quantum chemical calculations at the coupled-cluster level up to full quadruple excitations. To obtain a more accurate *A*_0_ value of the SO^+^ (X^2^Π_1/2, 3/2_), the complete basis set extrapolation, the zero-point vibrational energy correction, the core-valence electronic correction and the spin-orbit coupling corrections were included at the same time. A very accurate *A*_0_ value of 359.0 cm^−1^ for the SO^+^(X^2^Π_1/2, 3/2_) was obtained in their calculations.

As we know, both the core-valence correlation and scalar relativistic corrections have important effects on the accurate prediction of the spectroscopic parameters and molecular constants. On the one hand, as seen in previous theoretical work [9,10,13,22–25], only one [22] has included the core-valence correlation effect, and no results have taken into account the scalar relativistic correction. Therefore, to obtain more reliable spectroscopic and molecular properties, more work should be done so as to include the core-valence correlation and scalar relativistic corrections, in particular for the *A*_0_ calculations. On the other hand, the molecular properties of the SO^+^ ion have received little attention in the past several decades, whether in experiment or in theory. In addition, some vibrational levels in the ground state are missing and the vibrational levels for the A^2^Π electronic state are simply unknown in the past work. Therefore, there is room for improvement of the spectroscopic parameters by theory.

In the present work, the PECs of X^2^Π and A^2^Π electronic states of the SO^+^ molecular cation are calculated for internuclear separations from 0.08 to 1.06 nm. The calculations are performed using the complete active space SCF (CASSCF) method, which is followed by the internally contracted multi-reference CI (MRCI) approach [26,27] together with the correlation-consistent aug-cc-pV5Z (AV5Z) basis set [28–30]. Then, the effects on the PECs by the core-valence correlation and scalar relativistic corrections are included. To obtain more reliable PECs, the Davidson modification [31,32] based on the MRCI calculations (MRCI+Q) is taken into account. The spectroscopic parameters are obtained by fitting the vibrational levels, which are calculated by solving the ro-vibrational Schrödinger equation. The spectroscopic parameters are compared with those reported in the literature. Using the Breit-Pauli operator, the spin-orbit coupling effect on the spectroscopic parameters is included in the present PEC calculations of the X^2^Π and A^2^Π electronic states by two basis sets, aug-cc-pCVTZ (ACVTZ) and aug-cc-pVTZ (AVTZ) [33,34]. And finally, with the PECs obtained by the MRCI+Q/AV5Z+DK+CV calculations, the vibrational manifolds are calculated for each vibrational state of each electronic state, and those of the first 30 vibrational states are reported for the ^32^S^16^O^+^ cation for the *J* = 0 case. Comparison with the measurements demonstrates that the present results are much more accurate and reliable than the ones obtained by previous theoretical calculations.

## 2. Computational Details

Here we calculate the PECs of X^2^Π and A^2^Π electronic states of the SO^+^ cation by the CASSCF method, which is followed by the MRCI approach [26,27] for internuclear separations from 0.08 to 1.06 nm. Therefore, the full valence CASSCF is used as the reference wavefunction for the MRCI calculations in the present work. For the PEC calculations, the MRCI theory has proven particularly successful. Especially in recent years, we have reported a number of high-quality spectroscopic results for a variety of diatomic molecules [35–40]. Here, all the PEC calculations are performed using the MOLPRO 2008.1 program package [41].

MOLPRO only uses Abelian point group symmetry. For molecules with degenerate symmetry, an Abelian subgroup must be used. That is, for a diatomic cation such as SO^+^ with C_∞v_ symmetry, it will be substituted by C_2v_ symmetry with the order of irreducible representations being *a*_1_/*b*_1_/*b*_2_/*a*_2_. In the CASSCF and subsequent MRCI calculations, these four kinds of states would be evaluated. In detail, for the X^2^Π and A^2^Π electronic states of the SO^+^ cation, the eight valence MOs are put into the active space, including four *a*_1_, two *b*_1_ and two *b*_2_ symmetry MOs which correspond to the 3*p* shell of sulfur and 2*p* of oxygen atom. The rest of the electrons in the SO^+^ ion are put into six closed-shell orbitals, including four *a*_1_, one *b*_1_ and one *b*_2_ symmetry MOs. This results in a Complete Active Space (CAS) of 11 electrons in 8 orbitals, *i.e.*, CASSCF [8,11]. When we use the 14 MOs (8*a*_1_, 3*b*_1_ and 3*b*_2_) to make the PEC calculations of the X^2^Π and A^2^Π electronic states of the SO^+^ ion, we find that the PECs are smooth over the present internuclear separation range. Here, the main electronic configurations of the cation are 1σ^2^2σ^2^3σ^2^4σ^2^1Π^4^5σ^2^6σ^2^7σ^2^2Π^4^3Π^1^ for the X^2^Π and 1σ^2^2σ^2^3σ^2^4σ^2^1Π^4^ 5σ^2^6σ^2^7σ^2^2Π^3^3Π^2^ for the A^2^Π electronic state. In addition, for the present calculations, the SO^+^(X^2^Π) cation dissociates into the S^+^(^4^S_u_) atomic cation and O(^3^P_g_) atom, and the SO^+^(A^2^Π) cation dissociates into the S^+^(^2^D_u_) atomic cation and O(^3^P_g_) atom.

To accurately determine the PECs of the two electronic states, the interval used here is 0.02 nm, except near the equilibrium internuclear separation where the spacing is 0.002 nm. Here, the smaller step size is adopted around the equilibrium separation of each electronic state so that the properties of each PEC can be displayed more clearly.

With the aid of module VIBROT in the MOLCAS 7.4 program package [42], the spectroscopic parameters (excitation energy term *T_e_*, equilibrium internuclear separation *R_e_*, harmonic frequency *ω_e_*, first- and second-order anharmonic constants *ω_e_x_e_* and *ω_e_y_e_*, rotational constant *B_e_*, rotation-vibration coupling constant *α_e_* and rigid rotational constant *D_rot_*) and vibrational manifolds are calculated for the two electronic states. Here, we use the module VIBROT to make the corresponding vibration-rotation spectrum calculations. In the module VIBROT, the potential is fitted to an analytical form by cubic splines. The ro-vibrational Schrödinger equation is then solved by Numerov’s method [43]. That is, the ro-vibrational constants are calculated in a direct forward manner from the analytic potential by solving the ro-vibrational Schrödinger equation, and the spectroscopic parameters are determined by fitting the vibrational levels. Here, we collect the spectroscopic results obtained by the MRCI/AV5Z calculations in Table 1. In addition, we also present the experimental spectroscopic parameters reported in the literature [17] in the table for convenient comparison.

To include the effect on the spectroscopic results by the core-valence correlation corrections, we perform the PEC calculations of the two electronic states over the present internuclear separations by both taking and not taking into account the core-valence correlation effect using the ACVTZ basis set [33,34]. That is, the ACVTZ basis set with all electrons correlated and the ACVTZ basis set within the frozen-core approximation are used for the present core-valence correlation contribution calculations. Here, it should be pointed out that “all electrons correlated” for the sulfur atom do not include the two 1*s* electrons. And “within the frozen-core approximation” means that the 1*s*, 2*s* and 2*p* electrons of the sulfur and the 1*s* electrons of the oxygen atom are not correlated. In detail, for a given electronic state, the difference between the two energies yields the core-valence correlation contributions. Adding the core-valence correlation correction results to the present AV5Z values (denoted as +CV), we determine the PECs corrected by the core-valence correlation effect. We calculate the spectroscopic parameters with the aid of module VIBROT [42], and include the corresponding spectroscopic results in Table 1 for comparison.

To evaluate the effect on the spectroscopic parameters by the scalar relativistic correction, we perform the PEC calculations over the present internuclear separations at the level of a cc-pV5Z basis set by both taking and not taking into account the relativistic effect. In this work, we employ the third-order Douglas-Kroll Hamiltonian (DKH3) approximation [44–46] to make the present scalar relativistic correction calculations since the total energy at the DKH3 approximation can best yield the full 4-component scalar relativistic correction results. The cc-pV5Z-DK basis set [47] with the DKH3 approximation and the cc-pV5Z basis set with no scalar relativistic corrections are used for the scalar relativistic correction contribution calculations. In detail, for a given electronic state, the difference between the two energies yields the scalar relativistic correction results. Adding the scalar relativistic correction results to the present AV5Z values (denoted as +DK), we determine the PECs corrected by the relativistic effect. With the PECs obtained here, we calculate the spectroscopic results with the help of the module VIBROT [42]. Similar to those of the core-valence correlation correction, these spectroscopic parameters are also presented in Table 1 for comparison.

By simultaneously adding the core-valence correlation correction and scalar relativistic correction results determined above to the present AV5Z values, we obtain the PECs corrected by both effects. Using these PECs, the spectroscopic parameters are calculated with the aid of the module VIBROT [42]. The spectroscopic results are collected in Table 1 for comparison.

To obtain more reliable results, the PECs determined by the MRCI calculations are corrected for size-extensivity errors by means of the Davidson modification [31,32]. Similar to those in the MRCI calculations, we also include the additional core-valence correlation and/or scalar relativistic correction results in the present MRCI+Q/AV5Z values. It should be pointed out that the additional core-valence correlation and scalar relativistic corrections used here are calculated at the MRCI+Q level. With these PECs, we fit the spectroscopic parameters using the vibrational levels, which are obtained by solving the ro-vibrational Schrödinger equation with the aid of the module VIBROT [42]. The spectroscopic parameters determined here are collected in Table 1 for comparison.

To evaluate the effect on the *A*_0_ of the SO^+^(X^2^Π_1/2, 3/2_) and SO^+^(A^2^Π_1/2, 3/2_) by correlating core-valence electrons, we use two all-electron basis sets, ACVTZ and AVTZ, to investigate the spin-orbit coupling splitting of the two electronic states of the SO^+^ cation. The spin-orbit coupling calculations are performed by computing the Breit-Pauli spin-orbit matrix elements among the components of the interacting states using internally contracted MRCI wave functions [48], and the orbitals of involved Ω components are optimized by using the CASSCF approach. When we have obtained the PECs of the involved Ω components, the spectroscopic parameters are calculated with the aid of module VIBROT [42]. Adding the spin-orbit coupling corrections to the present AV5Z potential energies (denoted as +SO), we obtain the PECs corrected by the spin-orbit coupling effect. Adding the spin-orbit coupling corrections to the present AV5Z+CV+DK values (denoted as AV5Z+CV+DK+SO), we obtain the PECs corrected by the spin-orbit coupling, core-valence correlation and relativistic effects. With these PECs, the spectroscopic parameters of the involved Ω electronic states are evaluated with the aid of the same module VIBROT [42].

## 3. Results and Discussion

### 3.1. Spectroscopic Parameters of Λ-S States

The Davidson modification lowers the total energy by 26.084 and 29.531 mE_h_ for the X^2^Π and A^2^Π electronic states near the internuclear equilibrium separations, respectively. Table 1 demonstrates the effects on the *T_e_*, *R_e_*, *ω_e_* and other spectroscopic parameters by the Davidson modification. As seen in Table 1, (1) the effect on the *T_e_* of the A^2^Π electronic state by the Davidson modification is very significant. The shift of the *T_e_* lowered by the modification is 756.53 cm^−1^; (2) the Davidson modification lengthens the *R_e_* only by 0.00019 and 0.00013 nm for the X^2^Π and A^2^Π electronic states, respectively; (3) the effects on the *ω_e_* by the Davidson modification are unequal for the two electronic states. It lowers the *ω_e_* by 5.29 cm^−1^ for the X^2^Π but raises the *ω_e_* by 4.601 cm^−1^ for the A^2^Π electronic state. On the whole, the effects on the *T_e_* by the Davidson modification are more pronounced than those on the *R_e_* and *ω_e_*.

With only the core-valence correlation correction included in the X^2^Π and A^2^Π electronic states, the total energies are lowered by about 353.454 and 350.428 mE_h_ for the MRCI and 376.023 and 373.538 mE_h_ for the MRCI+Q calculations near the internuclear equilibrium separation, respectively. From Table 1, one can see that (1) the core-valence correlation correction makes the *T_e_* of the A^2^Π electronic state increase for the MRCI and MRCI+Q calculations; (2) the correlation correction shortens the *R_e_* of the X^2^Π and A^2^Π electronic states. In detail, the *R_e_* is shortened by 0.00043 and 0.00047 nm for the MRCI and 0.00039 and 0.00045 nm for the MRCI+Q calculations; (3) for the X^2^Π and A^2^Π electronic states, the correlation correction raises the *ω_e_* by 10.64 and 2.201 cm^−1^ for the MRCI and 9.92 and 3.152 cm^−1^ for the MRCI+Q calculations. On the whole, the effects on the *R_e_* and *ω_e_* by the core-valence correlation correction are more pronounced than those by the Davidson modification.

With only the scalar relativistic correction added in the X^2^Π and A^2^Π electronic states, the total energy is lowered by about 1.135 E_h_ near the internuclear equilibrium position. Table 1 collects the spectroscopic results corrected by the relativistic effect. As shown in Table 1, (1) the scalar relativistic correction lowers the *T_e_* of the A^2^Π electronic state by 63.65 cm^−1^ for the MRCI and 60.57 cm^−1^ for the MRCI+Q calculations; (2) the scalar relativistic correction has a very small effect on the *R_e_*. The largest shifts of *R_e_* are only 0.00001 and 0.00012 nm for the X^2^Π and A^2^Π electronic states, respectively; (3) for the X^2^Π electronic state, the scalar relativistic correction lowers the *ω_e_* by 2.76 and 2.71 cm^−1^ for the MRCI and MRCI+Q calculations. And for the A^2^Π electronic state, the scalar relativistic correction lowers the *ω_e_* by 2.087 and 1.975 cm^−1^ for the MRCI and MRCI+Q calculations. Obviously, the effects on the *T_e_*, *R_e_* and *ω_e_* by the scalar relativistic correction are smaller than those by the core-valence correlation correction.

With the core-valence correlation and scalar relativistic corrections included synchronously, one can find that the spectroscopic parameters are in excellent agreement with the measurements, in particular at the MRCI+Q level. For this reason, we make a brief comparison between the present results obtained by the MRCI+Q/AV5Z+DK+CV calculations and the measurements. (1) The present *T_e_* of the A^2^Π electronic state is 31429.43 cm^−1^, which is smaller than the measurements [17] by only 55.05 cm^−1^; (2) Favorable agreement can be found between the present *R_e_* results and the measurements [17]. The deviations of the present *R_e_* from the measurements [17] are 0.00030 (0.21%) and 0.00021 nm (0.13%) for the X^2^Π and A^2^Π electronic states; (3) Excellent agreement is observed between the present *ω_e_* and measurements. The deviations of the present *ω_e_* from the measurements [17] are 0.43 cm^−1^ for the X^2^Π and 0.94 cm^−1^ for the A^2^Π electronic state. (4) As shown in Table 1, other spectroscopic parameters (*ω_e_x_e_*, *B_e_*, *α_e_* and *D_rot_*) also agree favorably with the measurements [17]. The comparison demonstrates that the present calculations with the core-valence correlation and scalar relativistic corrections and Davidson modification can improve the quality of spectroscopic parameters. For convenient comparison, here we collect the spectroscopic results obtained by the MRCI+Q/AV5Z +CV+DK calculations together with the available experimental [10–15,17,20] and other theoretical [9,24,25] results in Table 2.

For the X^2^Π electronic state, as shown in Table 2, no other theoretical spectroscopic parameters are superior to the present ones when compared with the measurements [17]. In this respect, we think that the spectroscopic parameters of the SO^+^(X^2^Π) cation collected in Table 2 are of high quality.

By the way, at the MRCI+Q/AV5Z+CV+DK level, we have determined the dissociation energies, 5.4010 and 3.3976 eV, for the X^2^Π and A^2^Π Λ-S states, respectively. The experimental dissociation energy of the X^2^Π Λ-S state reported in [49] is 5.43 ± 0.19 eV, and the experimental dissociation energy of the A^2^Π Λ-S state is 3.3756 ± 0.19 eV if we employ the *T_e_* reported in [17]. Obviously, excellent agreement exists between the present dissociation energies and the experimental ones.

### 3.2. Spin-Orbit Effects in X^2^Π and A^2^Π States

For detailed comparison with available experimental and theoretical results, we study the effect on the spectroscopic parameters of the X^2^Π electronic state by the spin-orbit coupling correction. Lam *et al*. [22] in 2011 used the MRCI/cc-pwCV5Z method to calculate the *A*_0_ for the SO^+^(X^2^Π_1/2, 3/2_). Their result, 359.0 cm^−1^, is closer to the measurements [22] than the one, 330 cm^−1^, obtained by their MRCI calculations without using core-valence basis sets and all electrons (except two 1 s^2^ electrons of sulfur atom) in the active space. According to their theoretical results, Lam *et al*. [22] thought that the quality of the *A*_0_ could be improved by the additional treatment of core electrons. In addition, Lam *et al*. [22] also thought that it was premature at this point to conclude that correlating core electrons (augmented with appropriate core-valence basis sets) in the active space was a necessity for increasing the accuracy of the spin-orbit coupling calculations. To check this standpoint, here, we use two all-electron basis sets, ACVTZ and AVTZ, to perform the present spin-orbit coupling calculations. For the X^2^Π_1/2_, X^2^Π_3/2_, A^2^Π_1/2_ and A^2^Π_3/2_ Ω states, we collect the spectroscopic parameters obtained by the MRCI+Q/AV5Z+SO calculations in Table 3, for which the AVTZ basis set is used to calculate the spin-orbit coupling corrections; and we tabulate the spectroscopic parameters obtained by the MRCI+Q/AV5Z +SO calculations in Table 4, for which the ACVTZ basis set is used to calculate the spin-orbit coupling corrections. For convenient comparison, for the X^2^Π and A^2^Π Λ-S states, we present the spectroscopic parameters calculated by the MRCI+Q method in combination with the AV5Z basis set in Tables 3 and 4, respectively, for which the spin-orbit coupling corrections are omitted.

When the AVTZ basis set is used to perform the spin-orbit coupling calculations at the MRCI+Q level, the total energy of the X^2^Π_1/2_ component is −472.493589 E_h_, and the total energy of the X^2^Π_3/2_ component is −472.492020 E_h_ at the internuclear equilibrium position. The former is lower than and the latter is higher than the corresponding one, −472.492804 E_h_, of the X^2^Π electronic state. With the spin-orbit coupling correction added in the present MRCI+Q/AV5Z calculations, the energy separation of the two splitting components (X^2^Π_1/2_ and X^2^Π_3/2_) is 344.36 cm^−1^. According to the potential energies given here, it is not difficult to determine that the ground-state energy is lowered by about 172.29 cm^−1^ due to the spin-orbit coupling effect. As shown in Table 3, the spin-orbit coupling correction has no effect on the *R_e_* and only produces a very small effect on the *ω_e_*.

When the ACVTZ basis set is used to make the spin-orbit coupling calculations at the MRCI+Q level, the total energy of the X^2^Π_1/2_ component is −472.829051 E_h_, and the total energy of the X^2^Π_3/2_ component is −472.827401 E_h_ at the equilibrium position. At this time, the total energy of the SO^+^(X^2^Π) cation obtained by the MRCI+Q/ACVTZ calculations is −472.828226 E_h_. From this data, it is not difficult to determine that the *A*_0_ value of the SO^+^(X^2^Π_1/2, 3/2_) is 361.91 cm^−1^, and the ground-state energy of the cation is lowered by about 181.07 cm^−1^ due to the spin-orbit coupling effect. As shown in Table 4, the spin-orbit coupling correction has no effect on the *R_e_* and produces a very small effect on the *ω_e_*. For the X^2^Π electronic state, by comparison, it can be concluded that the ACVTZ basis set makes the spin-orbit coupling constant *A*_0_ of the SO^+^(X^2^Π_1/2, 3/2_) slightly larger and closer to the measurements [22] when compared with the one, AVTZ, for the spin-orbit coupling calculations.

Now we study the effect on the spectroscopic parameters by the spin-orbit coupling splitting when the core-valence correlation and scalar relativistic corrections are added. At this time, for the X^2^Π_1/2_, X^2^Π_3/2_, A^2^Π_1/2_ and A^2^Π_3/2_ Ω states, the spectroscopic results obtained by using the AVTZ basis set for the spin-orbit coupling calculations are presented in Table 5. Similar to Tables 3 and 4, here we also tabulate the spectroscopic results obtained by the MRCI+Q/AV5Z+CV+DK calculations without the spin-orbit coupling in Table 5 as the X^2^Π and A^2^Π results for comparison. By comparison between Table 3 and 5, we find that the inclusion of core-valence correlation and scalar relativistic corrections does not bring about the effect on the *A*_0_, but makes the *R_e_* and *ω_e_* closer to the measurements [12,13].

**Figure 1:**
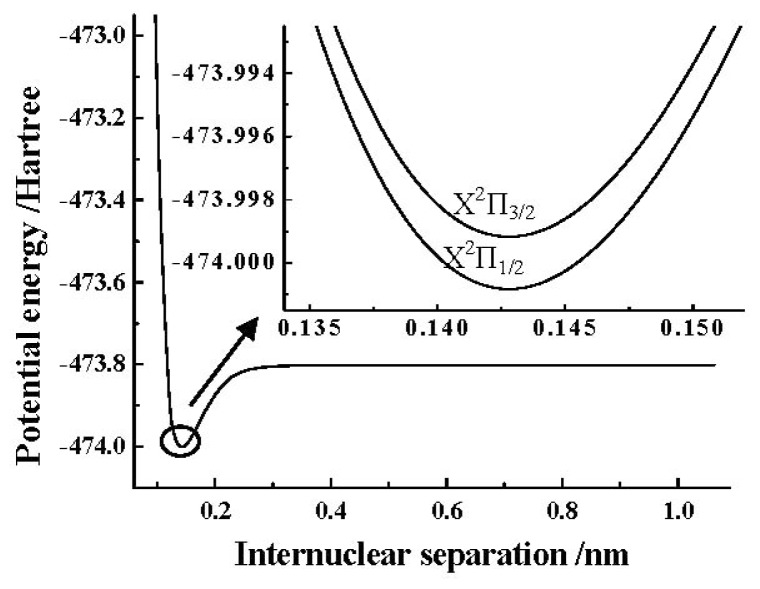
Potential energy curves (PECs) of the SO^+^(X^2^Π) and its two components near the equilibrium position.

Table 6 presents the spectroscopic parameters of the X^2^Π_1/2_ and X^2^Π_3/2_ components obtained by the MRCI+Q/AV5Z+CV+DK+SO calculations. Different from Table 5, it should be pointed out that the spin-orbit coupling calculations in Table 6 are performed with the core-valence correlation ACVTZ basis set. Here, we tabulate the spectroscopic parameters obtained by the MRCI+Q/AV5Z+CV+DK calculations without the spin-orbit coupling in Table 6 as the “X^2^Π” results, and we also collect some experimental [10–13] and theoretical [10,13,23] results in Table 6 for convenient comparison. In order to avoid congestion in Table 6, other experimental [14,15,17,18,20,22] and theoretical [9,13,22] *A*_0_ values of the SO^+^(X^2^Π_3/2, 1/2_) are presented in Table 7. The PEC obtained by the MRCI+Q/AV5Z+CV+DK calculations of the SO^+^(X^2^Π) cation is depicted in Figure 1. In addition, the detailed PECs of the SO^+^(X^2^Π_3/2, 1/2_) components near the equilibrium position obtained by using the ACVTZ basis set for the spin-orbit coupling corrections are also shown in the same Figure 1.

As seen in Table 6, at the MRCI+Q/AV5Z+CV+DK+SO level, the *A*_0_ of the SO^+^(X^2^Π_1/2, 3/2_) obtained by using the ACVTZ basis set for the spin-orbit coupling calculations is 362.13 cm^−1^, which agrees well with the recent measurements, 365.36 cm^−1^ [22]. The result is obviously superior to the one obtained by using the AVTZ basis set for the spin-orbit coupling correction. As demonstrated in Table 7, the *A*_0_ difference between the ACVTZ and AVTZ basis set is 17.77 cm^−1^. The conclusion can also be drawn that the ACVTZ basis set makes the *A*_0_ of the SO^+^(X^2^Π_1/2, 3/2_) slightly larger and closer to the measurements [22] when compared with the one, AVTZ, for the spin-orbit coupling calculations.

From Tables 6 and 7, at the MRCI+Q/AV5Z+CV+DK+SO level, we can clearly see that the *A*_0_ of the SO^+^(X^2^Π_1/2, 3/2_) obtained by using the ACVTZ basis set for the spin-orbit coupling calculations is the closest to the recent measurements [22] among all the theoretical results [9,10,13,22]. Other spectroscopic results such as *R_e_*, *ω_e_* and *ω_e_x_e_* also agree favorably with the experimental ones [12,13]. As a conclusion, we think that the spectroscopic parameters collected in Table 6 are of high quality.

As shown in Table 2, only Houria *et al*. [9] in 2006 have studied the spectroscopic parameters of the A^2^Π electronic state. Obviously, the present results are superior to those obtained by Houria *et al*. [9] when compared with the measurements [17].

At the equilibrium position, when the AVTZ basis set is used to calculate the spin-orbit coupling splitting, we find that the total energy of A^2^Π_1/2_ component is higher, but the total energy of A^2^Π_3/2_ is lower than that of the SO^+^(A^2^Π) cation. With the correction results added into the present MRCI+Q/AV5Z values, the obtained spectroscopic results are collected in Table 3. As shown in Table 3, the *A*_0_ for the SO^+^(A^2^Π_3/2, 1/2_) is 52.67 cm^−1^, which is in excellent agreement with the experimental one, 53.88 cm^−1^ [17]. At this time, the effects on the *R_e_* and *ω_e_* by the spin-orbit coupling are still very small, and the separations between the A^2^Π_1/2_ and A^2^Π_3/2_ components are only 0.00001 nm for the *R_e_* and 0.65 cm^−1^ for the *ω_e_*.

At the equilibrium position, when the ACVTZ basis set is used to calculate the spin-orbit coupling splitting, we also find that the total energy of the A^2^Π_1/2_ component is higher, and the total energy of A^2^Π_3/2_ is lower than that of the SO^+^(A^2^Π) cation. With these correction results added into the present MRCI+Q/AV5Z values, the obtained spectroscopic results are collected in Table 4. As shown in Table 4, the *A*_0_ for the SO^+^(A^2^Π_3/2, 1/2_) is 57.94 cm^−1^, which deviates more [22] than the one, 52.67 cm^−1^, obtained by using the AVTZ basis set for the spin-orbit coupling calculations. In addition, the effects on the *R_e_* and *ω_e_* by the spin-orbit coupling correction are very small, and the separations between the A^2^Π_3/2, 1/2_ components are only 0.00003 nm for the *R_e_* and 0.789 cm^−1^ for the *ω_e_*, respectively.

Table 5 also tabulates the spectroscopic results obtained by using the AVTZ basis set for the spin- orbit coupling calculations of the A^2^Π_1/2_ and A^2^Π_3/2_ components at the MRCI+Q/AV5Z+CV+DK level. As shown in Table 5, the inclusion of core-valence correlation and scalar relativistic corrections brings about no effect on the *A*_0_ for the SO^+^(A^2^Π_3/2, 1/2_), and still produces a very small effect on the *R_e_* and *ω_e_*.

**Figure 2:**
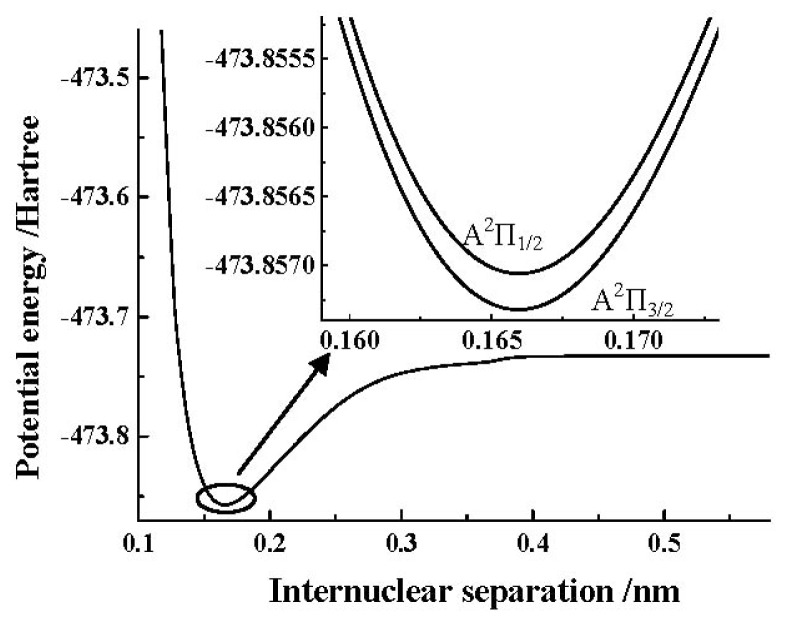
PECs of the SO^+^(A^2^Π) and its two components near the equilibrium position.

Table 6 demonstrates the effect on the spectroscopic results of the A^2^Π_1/2_ and A^2^Π_3/2_ components by the core-valence correlation and scalar relativistic corrections when the ACVTZ basis set is used to make the spin-orbit coupling correction calculations. One can still find that the effects on the *A*_0_, *R_e_* and *ω_e_* by the spin-orbit coupling correction are very small. In addition, we depict the PEC obtained by the MRCI+Q/AV5Z+CV+DK calculations of the SO^+^(A^2^Π) cation in Figure 2. Similar to the X^2^Π Λ-S state, to clearly show the details of the spin-orbit coupling splitting, we also depict the PECs obtained by the MRCI+Q/AV5Z+CV+DK+SO calculations of SO^+^(A^2^Π_3/2, 1/2_) components using the ACVTZ basis set for the spin-orbit coupling correction in the same Figure 2.

As a conclusion, we think that (1) for the MRCI+Q/AV5Z+CV+DK+SO calculations, the *A*_0_ of the SO^+^(X^2^Π_1/2, 3/2_) obtained by the ACVTZ basis set is closest to the measurements [22]. The *A*_0_ of the SO^+^(A^2^Π_1/2, 3/2_) obtained by the ACVTZ basis set also agree well with the measurements [17], and the difference between such *A*_0_ result and the experimental one is only several cm^−1^; (2) the core-valence correlations make the *A*_0_ become large for the two electronic states but are not sure to increase the accuracy of the spin-orbit coupling constant *A*_0_; (3) the spectroscopic results determined by the MRCI+Q/AV5Z+CV+DK calculations for the X^2^Π and A^2^Π electronic states have achieved a high quality.

### 3.3. Vibrational Manifolds

Here, we only use the PECs obtained by the MRCI+Q/AV5Z+DK+CV calculations to determine the vibrational manifolds of X^2^Π and A^2^Π electronic states. The reason is that no spin-orbit coupling experimental *G*(*υ*), *B_υ_* and *D_υ_* values exist in the literature, whereas the corresponding results can be found for the X^2^Π and A^2^Π electronic states. The vibrational level *G*(*υ*), inertial rotation constant *B_υ_* and centrifugal distortion constant *D_υ_* are predicted for each vibrational state of each electronic state by solving the ro-vibrational Schrödinger equation of nuclear motion using Numerov’s method [43]. Due to length limitation, here we only tabulate the *G*(*υ*), *B_υ_* and *D_υ_* results of the first 30 vibrational states of SO^+^(X^2^Π) and SO^+^(A^2^Π) cation for the *J* = 0 case in Tables 8 and 9, respectively.

For the *G*(*υ*) of X^2^Π electronic state, only one group of RKR data can be found in the literature [49]. We collect the only group of RKR data in Table 8 for comparison. As seen in Table 8, excellent agreement exists between them. For example, the deviations of the present *G*(*υ*) results from the RKR data [49] is only 0.29, 3.67, 6.45 and 8.97 cm^−1^ for *υ* = 0, 6, 10 and 16, respectively.

At least four groups of *B_υ_* experimental data exist in the literature [15–17,21] for the SO^+^(X^2^Π) cation. In order to avoid congestion in Table 8, here we only tabulate the *B_υ_* given by Hardwick *et al*. [15], Milkman *et al*. [16] and Dyke *et al*. [21] for comparison. As demonstrated in Table 8, the present *B_υ_* are in excellent agreement with all the measurements [15,16,21] collected in Table 1. For example, the largest deviation of the present *B_υ_* results from the measurements [15] is 0.34% (which corresponds to *υ* = 4). The largest deviation of the present *B_υ_* results from the measurements [16] is 0.373% (which corresponds to *υ* = 4). And the largest deviation of the present *B_υ_* results from the measurements [21] is 0.34%. When we compare the present *B_υ_* with those [17] not collected in Table 8, good accord also exists between them. Therefore, we think, with reason, that the newly calculated *B_υ_* results are of a very high quality.

Similar to the *B_υ_*, there are also four groups of measurements [15–17,21] and one group of RKR data [17] concerning the *D_υ_* of the SO^+^(X^2^Π) cation. To avoid congestion in Table 8, we only tabulate three groups of measurements [15,16,21] and one group of RKR data in the table. It is not difficult to find that excellent agreement exists between the present results and the measurements [15,16] as well as RKR data [17]. For example, the present results are smaller than the measurements [15] only by 0.79% and 3.85% for *υ* = 4 and 5, and the present results are smaller than the experimental data [21] also only by 0.76% and 1.74% for *υ* = 0 and 1, respectively. Because the *D_υ_* is a very small quantity, such deviation is acceptable. In addition, when we compare the experimental *D_υ_* results [17] not collected in Table 8, excellent agreement can also be found between them.

Table 9 collects the present *G*(*υ*), *B_υ_* and *D_υ_* results of the ^32^S^16^O^+^(A^2^Π) cation until *υ* = 29 together with three groups of measurements [15–17]. From Table 9, we can see that the difference between the G(0) and G(1) is equal to 792.2 cm^−1^, whereas the corresponding experimental difference obtained by Coxon and Foster [14] is 792.7 cm^−1^. Excellent agreement exists between the present result and the experimental one. As seen in Table 9, the present *B_υ_* results agree favorably with the measurements [15–17]. For example, the differences between the present *B_υ_* results and the measurements [15] are only 0.17% and 0.19% for *υ* = 0 and 1, and the differences between the present *B_υ_* and the measurements [16] are 0.12%, 0.30%, 0.04% and 0.49% for *υ* = 0, 4, 7 and 11, respectively. At the same time, the largest deviation of the present *B_υ_* results from the measurements [17] is also only by 0.29% (which corresponds to *υ* = 5). All the comparisons demonstrate that the present *B_υ_* results tabulated in Table 9 are accurate.

As for the *D_υ_* results of the ^32^S^16^O^+^(A^2^Π) cation, three groups of experimental results [15–17] and one group of RKR data [17] have been found in the literature to our knowledge. For convenient comparison with the present results and to avoid congestion, only some of these experimental data are collected in Table 9. As seen in Table 9, excellent agreement with the measurements [15,16] and the RKR data [17] still exists. For example, the largest deviation of the present *D_υ_* from the measurements [15] is only by 0.60%, and the differences between the present *D_υ_* and the RKR data [17] are also only 0.31%, 0.23%, 0.74% and 0.56% for *υ* = 0, 4, 7 and 11, respectively. As noted above, the *D_υ_* is a very small quantity. Anyway, such deviation is still very small.

To the best of our knowledge, no *G*(*υ*) results can be found in the literature for the ^32^S^16^O^+^(A^2^Π) ion, either theoretically or experimentally. Therefore, we cannot make any direct comparison between them. On the one hand, as seen in Tables 2 and 6, the present spectroscopic parameters obtained by the MRCI+Q/AV5Z+CV+DK calculations agree well with the measurements for the two electronic states. On the other hand, the vibrational manifolds of the ground state and the *B_υ_* and *D_υ_* results of the A^2^Π electronic state are also in excellent agreement with the experimental data. Because all the results are calculated by the same approach and fitted by the same procedure, we believe that the *G*(*υ*) results of the A^2^Π electronic state collected in Table 9 and the vibrational manifolds for higher vibrational levels presented in Tables 8 and 9 are reliable and accurate. They should be of considerable value for future experimental or theoretical research.

Finally, we will discuss the effect on the vibrational manifolds by the spin-orbit coupling correction [50–53]. On the whole, the spin-orbit coupling correction brings about only small change for lower *G*(*υ*), whereas it can produce the shift of more than ten cm^−1^ for higher *G*(*υ*). For example for the X^2^Π electronic state, the *G*(3) is 3,218.42 cm^−1^ for the X^2^Π_1/2_ and 4,476.08 cm^−1^ for the X^2^Π_3/2_, respectively, which deviate from the *G*(3) only by 1.35 cm^−1^. And the *G*(29) is 31,682.47 cm^−1^ for the X^2^Π_1/2_ and 31,644.37 cm^−1^ for the X^2^Π_3/2_, respectively, which deviate from the *G*(29) by 19.07 cm^−1^.

## 4. Conclusions

In this work, the PECs of the X^2^Π and A^2^Π electronic states of the SO^+^ cation have been studied employing the CASSCF method followed by the MRCI approach in combination with the correlation-consistent AV5Z basis set for internuclear separations from 0.08 to 1.06 nm. The effects on the PECs by the core-valence correlation and scalar relativistic corrections have been included. Scalar relativistic corrections are made using the DKH3 approximation at the level of a cc-pV5Z basis set. Core-valence correlation corrections are included with a cc-pCVTZ basis set. To obtain more reliable results, the PECs obtained by the MRCI calculations are corrected for size-extensivity errors by means of Davidson modification. The effects on the spectroscopic parameters by the spin-orbit coupling splitting are included using the Breit-Pauli operator with two all-electron basis sets, AVTZ and ACVTZ. With the PECs obtained here, the spectroscopic parameters of the two electronic states have been obtained by fitting the vibrational levels, which are calculated by solving the ro-vibrational Schrödinger equation with Numerov’s method. The spectroscopic parameters obtained by the MRCI+Q/AV5Z+CV+DK calculations have been found tob e in excellent agreement with the experimental results. At the MRCI+Q/AV5Z+CV+DK level, the *A*_0_ of the SO^+^(X^2^Π_1/2, 3/2_) and SO^+^(A^2^Π_1/2, 3/2_) are 362.13 and 58.16 cm^−1^ when the ACVTZ basis set is used for the spin-orbit coupling calculations, and the *A*_0_ of the SO^+^ (X^2^Π_1/2, 3/2_) and SO^+^(A^2^Π_1/2, 3/2_) are 344.36 and 52.90 cm^−1^ when the AVTZ basis set is used for the spin-orbit coupling calculations. We conclude that the core-valence correlation ACVTZ basis set makes the *A*_0_ slightly large when compared with the AVTZ set, which does not correlate core-valence electrons. With these PECs determined by the MRCI+Q/AV5Z+CV+DK calculations, the vibrational manifolds are calculated for each vibrational state of the two electronic states, and those of the first 30 vibrational states are reported for the ^32^S^16^O^+^ cation for the *J* = 0 case. Comparison with the experimental results demonstrates that the present vibrational manifolds are both reliable and accurate.

## Figures and Tables

**Table 1:** Effect on the spectroscopic parameters of the ^32^S^16^O^+^ ion by the core-valence correlation and/or scalar relativistic corrections at the AV5Z basis set.

	*T_e_*/cm^−1^	*R_e_*/nm	*ω_e_*/cm^−1^	*ω_e_x_e_*/cm^−1^	10^3^*ω_e_y_e_*/cm^−1^	*B_e_*/cm^−1^	*α_e_*/cm^−1^	10^6^*D_rot_*/cm^−1^
**X^2^Π**								
MRCI	0	0.14295	1,304.42	7.70970	1.38485	0.773776	6.08585	1.08929
+DK	0	0.14295	1,301.66	7.69388	1.76821	0.773702	6.09391	1.09362
+CV	0	0.14252	1,315.06	7.74244	1.94077	0.778422	6.09517	1.09226
+DK+CV	0	0.14253	1,312.31	7.72776	1.80150	0.778366	6.10304	1.09133
MRCI+Q	0	0.14314	1,299.13	7.71239	1.19810	0.771652	6.08708	1.08677
+DK	0	0.14315	1,296.42	7.70541	2.47700	0.771582	6.09563	1.09251
+CV	0	0.14275	1,309.05	7.75005	0.64338	0.775972	6.10065	1.08836
+DK+CV	0	0.14275	1,306.35	7.74283	1.24649	0.775919	6.10957	1.09664
Exp. [17]	0	0.14245	1,306.78	7.6975	1.90	0.778592	6.2100	
**A^2^Π**								
MRCI	31,640.56	0.16615	798.904	6.36511	0.38008	0.573152	6.29707	1.09776
+DK	31,576.91	0.16623	796.817	6.32371	0.61314	0.572579	6.27247	1.10141
+CV	32,304.69	0.16568	801.105	6.35370	4.37020	0.576501	6.49154	1.09387
+DK+CV	32,239.07	0.16576	798.969	6.31014	4.20088	0.575924	6.46491	1.09793
MRCI+Q	30,884.03	0.16628	803.505	6.45960	6.75393	0.572299	6.18139	1.08717
+DK	30,823.46	0.16636	801.530	6.42811	6.76501	0.571758	6.16112	1.09064
+CV	31,429.43	0.16583	806.657	6.52598	1.02596	0.575518	6.37255	1.08261
+DK+CV	31,366.44	0.16591	804.634	6.49255	0.98191	0.574975	6.35074	1.08654
Exp. [17]	31,421.49	0.16570	805.594	6.507	3.1	0.57534	5.9137	

**Table 2:** Comparison of the spectroscopic parameters obtained by the MRCI+Q/AV5Z+CV+DK calculations with measurements and other theoretical results.

	*T_e_*/cm^−1^	*R_e_*/nm	*ω_e_*/cm^−1^	*ω_e_x_e_*/cm^−1^	10^3^*ω_e_y_e_*/cm^−1^	*B_e_*/cm^−1^	10^3^*α_e_*/cm^−1^	10^6^*D_rot_*/cm^−1^
**X^2^Π**								
This work	0	0.14275	1,306.35	7.74283	1.24649	0.77599	6.10957	1.09664
Exp. [10]	0	0.142(4) (a)	1,360 ± 30					
Exp. [14]	0	0.14238	1,307.15	7.741	---	0.7800	6.31	1.04
Exp. [15]	0	0.14250	1,311.44	8.365	29	0.7787	6.224	1.02
Exp. [17]	0	0.14245	1,306.78	7.6975	1.90	0.77859	6.2100	
Exp. [20]	0	---	1,330 ± 30	8.0 ± 6.0				
Cal. [9] (b)	0	0.1434	1,305.5	9.02	150	0.769	7	
Cal. [24] (c)	0	0.1411						
Cal. [25] (d)	0	0.1421	1,359					
**A^2^Π**								
This work	31,366.44	0.16591	804.634	6.49255	0.98191	0.574975	6.35074	1.08654
Exp. [10]	32,593							
Exp. [11]	32,943	---	805 ± 5	6.4 ± 0.5				
Exp. [12]	31,633 ± 10	---	804.4 ± 1.6	6.34 ± 0.18				
Exp. [13] (e)	---	0.1663	805	6.4				
Exp. [14]	31,422.75	0.16570	805.36	6.34	---	0.5759	5.82	1.17
Exp. [15]	31,432	0.16578	805.25	6.34	---	0.57536	5.88	1.17
Exp. [17]	31,421.49	0.16570	805.594	6.507	3.1	0.57534	5.9137	
Cal. [9] (b)	30,439.9	0.1670	786.6	6.41	−50	0.567	5	

(a) 0.142 nm is of *r*_0_ value, not *r_e_*;

(b) these results were calculated by the MRCI+Q/cc-pV5Z approach in Ref. [9];

(c) these results were calculated by the SCF/6-31G*(5*d*) approach in Ref. [24];

(d) these results were calculated by the HF/DF B3LYP/6-311++G(3*df*, 3*pd*) approach in Ref. [25];

(e) 0.1663 nm is of *r*_0_ value, not *r_e_*.

**Table 3:** Spectroscopic parameters obtained by the MRCI+Q/AV5Z+SO calculations using the AVTZ basis set for the spin-orbit coupling corrections.

	*T_e_*/cm^−1^	*R_e_*/nm	*ω_e_*/cm^−1^	*ω_e_x_e_*/cm^−1^	10^3^*ω_e_y_e_*/cm^−1^	*B_e_*/cm^−1^	10^3^*α_e_*/cm^−1^	10^6^*D_rot_*/cm^−1^
**X^2^Π**	172.29	0.14314	1299.13	7.71239	1.19810	0.771652	6.08708	1.08677
**X^2^Π_1/2_**	0	0.14314	1299.45	7.70013	2.14436	0.771651	6.08277	1.08397
**X^2^Π_3/2_**	344.36	0.14314	1298.80	7.71643	2.05483	0.771653	6.09135	1.09157
**A^2^Π**	31,056.32	0.16628	803.505	6.45960	6.75393	0.572299	6.18139	1.08717
**A^2^Π_3/2_**	31,028.01	0.16628	803.855	6.41096	6.63778	0.572351	6.16617	1.08512
**A^2^Π_1/2_**	31,080.68	0.16629	803.205	6.50258	6.89782	0.572257	6.19580	1.08847

The *T_e_* value of the ^32^S^16^O^+^(X^2^Π_1/2_) component is set to zero; All other *T_e_* results (in Tables 3–6) are relative to the *T_e_* of the ^32^S^16^O^+^(X^2^Π_1/2_) component.

**Table 4:** Spectroscopic parameters obtained by the MRCI+Q/AV5Z+SO calculations using the ACVTZ basis set for the spin-orbit coupling corrections.

	*T_e_*/cm^−1^	*R_e_*/nm	*ω_e_*/cm^−1^	*ω_e_x_e_*/cm^−1^	10^3^*ω_e_y_e_*/cm^−1^	*B_e_*/cm^−1^	10^3^*α_e_*/cm^−1^	10^6^*D_rot_*/cm^−1^
**X^2^Π**	181.07	0.14314	1,299.13	7.71239	1.19810	0.771652	6.08708	1.08677
**X^2^Π_1/2_**	0	0.14314	1,299.51	7.70738	2.12502	0.771655	6.08272	1.08485
**X^2^Π_3/2_**	361.91	0.14314	1,298.77	7.72159	1.90197	0.771649	6.09184	1.09000
**A^2^Π**	31,065.10	0.16628	803.505	6.45960	6.75393	0.572299	6.18139	1.08717
**A^2^Π_3/2_**	31,033.93	0.16627	803.929	6.40703	6.64887	0.572373	6.16694	1.08487
**A^2^Π_1/2_**	31,091.87	0.16630	803.140	6.50548	6.95249	0.572234	6.19544	1.08870

**Table 5:** Spectroscopic results obtained by the MRCI+Q/AV5Z+CV+DK+SO calculations using the AVTZ basis set for the spin-orbit coupling corrections.

	*T_e_*/cm^−1^	*R_e_*/nm	*ω_e_*/cm^−1^	*ω_e_x_e_*/cm^−1^	10^3^*ω_e_y_e_*/cm^−1^	*B_e_*/cm^−1^	10^3^*α_e_*/cm^−1^	10^6^*D_rot_*/cm^−1^
**X^2^Π**	172.29	0.14275	1,306.35	7.74283	1.24649	0.775919	6.10957	1.09664
**X^2^Π_1/2_**	0	0.14275	1,306.65	7.73061	1.58431	0.775916	6.10511	1.09427
**X^2^Π_3/2_**	344.36	0.14275	1,306.03	7.74804	1.63230	0.775922	6.11356	1.09741
**A^2^Π**	31,538.72	0.16591	804.634	6.49255	0.98191	0.574975	6.35074	1.08654
**A^2^Π_3/2_**	31,510.19	0.16590	804.986	6.44634	0.82722	0.575028	6.33707	1.08485
**A^2^Π_1/2_**	31,563.09	0.16591	804.332	6.53314	1.17497	0.574932	6.36382	1.08810

**Table 6:** Spectroscopic results obtained by the MRCI+Q/AV5Z+CV+DK+SO calculations using the ACVTZ basis set for the spin-orbit coupling corrections.

	*T_e_*/cm^−1^	*R_e_*/nm	*ω_e_*/cm^−1^	*ω_e_x_e_*/cm^−1^	10^3^*ω_e_y_e_*/cm^−1^	*B_e_*/cm^−1^	10^3^*α_e_*/cm^−1^	10^6^*D_rot_*/cm^−1^
**X^2^Π**	181.07	0.14275	1,306.35	7.74283	1.24649	0.775919	6.10957	1.09664
**X^2^Π_1/2_**	0	0.14275	1,306.71	7.73820	1.59213	0.775920	6.10524	1.09634
Exp. [11]	0	---	1,323 ± 3	7.8 ± 0.3				
Exp. [12]	0	---	1,307.5 ± 1.9	7.84 ± 0.21				
Exp. [13]	0	0.1424	1,307	7.75	---	0.771	6.3	
Cal. [13]	0	0.1453	1,270	8.0				
**X^2^Π_3/2_**	362.13	0.14275	1,306.00	7.75207	1.35461	0.775919	6.11411	1.09714
Exp. [10]	340 ± 25							
Exp. [11]	414 ± 5	---	1,323 ± 3	7.8 ± 0.3				
Exp. [12]	412 ± 13	---	1,307.5 ± 1.9	7.84 ± 0.21				
Exp. [13]	352	0.1424	1,307	7.75	---	0.781	6.3	
Cal. [10]	360 a							
Cal. [23]		339.2 b, 328 c						
**A^2^Π**	31,547.50	0.16591	804.634	6.49255	0.98191	0.574975	6.35074	1.08654
**A^2^Π_3/2_**	31,516.12	0.16589	805.062	6.44288	0.82556	0.575050	6.33605	1.08463
Exp. [13]	30,910	0.1663 d	805	6.4		0.567 e		
**A^2^Π_1/2_**	31,574.28	0.16592	804.265	6.53567	1.23183	0.54908	6.36317	1.08833
Exp. [13]	30,982	0.1663 d	805	6.4		0.575^e^		
Cal. [13]	30,600	0.1685	912	2.6				

a such *T_e_* value was obtained by the restricted CNDO calculations;

b such *T_e_* value was obtained by the MRD-CI/basis set 2;

c such *T_e_* value was obtained by the MRD-CI/basis set 4;

d these values are of *r*_0_, not *r_e_*;

e: these values are of *B*_0_, not *B_e_*.

**Table 7:** Comparison of the present spin-orbit coupling constant with the experimental and other theoretical results.

	This work a	This work b	Exp. [14]	Exp. [15]	Exp. [17]	Exp. [18]	Exp. [20]	Exp. [22]	Cal. [9]	Cal. [13]	Cal. [22]
**X^2^Π**	362.13	344.36	367.18	363.8	364.38	371 ± 20	355 ± 30	365.36	330.5 ± 20	338	359.0
**A^2^Π**	58.16	52.90	53.22	53.91	53.880	---	---	---	54.6	62	

a Spin-orbit coupling splitting is calculated by using the MRCI+Q method and the ACVTZ basis set;

b Spin-orbit coupling splitting is calculated by using the MRCI+Q method and the AVTZ basis set.

**Table 8:** Comparison of the present *G*(*υ*), *B_υ_* and *D_υ_* results with the experimental ones for the ^32^S^16^O^+^(X^2^Π) cation for the *J* = 0 case.

*υ*	*G*(*υ*)/cm^−1^		*B_υ_*/cm^−1^			10^6^*D_υ_*/cm^−1^			
	
	This work	Exp. [49]	This work	Exp. [15]	Exp. [16]	This work	Exp. [15]	Exp. [16]	Exp. [17]
0	651.27	651.56	0.772863	0.775508 ^[21]^	0.77548	1.09747	1.10591 ^[21]^	1.0941	1.1072
1	1,942.10	---	0.766739	0.769312 ^[21]^	0.76815	1.10154	1.121 ^[21]^	---	1.1107
2	3,217.48	3,219.06	0.760600	---	0.76219	1.10576	---	---	1.1146
3	4,477.42	---	0.754449	---	0.75674	1.11040	---	---	1.1189
4	5,721.89	5,724.56	0.748283	0.75087	0.75109	1.11513	1.124	1.39	1.1238
5	6,950.89	---	0.742100	0.744468	0.74446	1.12016	1.165	1.13	1.1291
6	8,164.39	8,168.06	0.735899	0.738269	0.73826	1.12565		1.16	1.1350
7	9,362.35	---	0.729677	0.73200	0.73171	1.13145		0.96	1.1414
8	10,544.75	10,549.56	0.723435	0.72618	0.72604	1.13802		1.26	1.1485
9	11,711.53	---	0.717171	0.71959	0.71953	1.14556		1.18	1.1562
10	12,862.61	12,869.06	0.710889		0.71281	1.15441			
11	13,997.89		0.704589			1.16486			
12	15,119.26	15,126.56	0.698281			1.17806			
13	16,220.59	---	0.691991			1.27147			
14	17,317.68	17,322.06	0.685758			1.22832			
15	18,378.15	---	0.679634			1.27147			
16	19,446.59	19,455.56	0.673705			1.32470			
17	20,467.74		0.668044			1.38496			
18	21,486.49		0.662680			1.44558			
19	22,488.00		0.657592			1.50143			
20	23,472.55		0.652694			1.54575			
21	24,440.60		0.647918			1.57178			
22	25,392.80		0.643209			1.58726			
23	26,329.83		0.638524			1.59627			
24	27,252.32		0.633886			1.60812			
25	28,160.85		0.629318			1.62867			
26	29,055.84		0.624858			1.65654			
27	29,937.70		0.620519			1.68893			
28	30,806.76		0.0616294			1.7182			
29	31,663.40		0.612159			1.74116			

**Table 9:** Comparison of the present *G*(*υ*), *B_υ_* and *D_υ_* results with the experimental ones for the ^32^S^16^O^+^(A^2^Π) cation for the *J* = 0 case.

*υ*	*G*(*υ*)/cm^−1^	*B_υ_*/cm^−1^				10^6^*D_υ_*/cm^−1^			
			
		This work	Exp. [15]	Exp. [16]	Exp. [17]	This work	Exp. [15]	Exp. [16]	Exp. [17]
0	401.15	0.571455	0.572415	0.57241	0.572398	1.17445	1.177	1.179	1.1781
1	1,193.37	0.565457	0.566532	0.56653	0.566491	1.18482	1.192	1.196	1.1873
2	1,972.15	0.559429	0.56054	0.560580	1.19520			---	1.1970
3	2,737.53	0.553379	0.55422	0.554356	1.20529			0.781	1.2071
4	3,489.61	0.547317	0.54899	0.548720	1.21449			---	1.2173
5	4,228.53	0.541255	1.54313	0.542821	1.22274			1.458	1.2271
6	4,954.51	0.535211	1.53665		1.22934				1.2361
7	5,667.80	0.529198	0.5294		1.23839				1.2440
8	6,368.68	0.523234	0.52256	1.524587	1.23839				1.2503
9	7,057.50	0.517353	0.52142		1.23980				1.2543
10	7,734.69	0.511581	1.5157		1.24239				1.2555
11	8,400.64	0.505942	0.50349		1.24640				1.2534
12	9,055.74	0.500478			1.25584				
13	9,700.31	0.495210			1.27332				
14	10,334.63	0.490182			1.30041				
15	10,958.89	0.485407			1.34218				
16	11,573.16	0.480890			1.39659				
17	12,177.42	0.476613			1.45946				
18	12,771.62	0.472557			1.52312				
19	13,355.79	0.468642			1.58337				
20	13,929.90	0.464783			1.62798				
21	14,494.06	0.460898			1.66019				
22	15,048.36	0.456919			1.68194				
23	15,592.90	0.452844			1.70288				
24	16,127.75	0.448689			1.73583				
25	16,652.90	0.444502			1.77888				
26	17,168.38	0.440344			1.81741				
27	17,674.40	0.436361			1.83136				
28	18,171.59	0.432628			1.83638				
29	18,660.61	0.429075			1.84735				
